# Case of Transfusion of Blood

**Published:** 1851-09

**Authors:** 


### OBSTETRICS.

*Case of Transfusion of Blood.*—The following case of successful *Transfusion* is reported by Dr. Marmonier, in the *Gazette Médicale de Paris*, 5th July, 1851, and is copied into the *London Medical Times* 9th August, 1851, from which we extract it. It is particularly interesting from the simplicity and ease with which the operation was performed.

"Jan. 3, 1851.—At six o'clock in the morning I was called to a woman named Mallet, of Lancey, aged 30 years, of a lymphatic constitution, somewhat weakened by many pregnancies occurring in quick succession, by previous difficult labors, and by moral and physical trials. The labor pains on my arrival had nearly ceased. The patient was weak and exhausted from long-continued and useless efforts, which were unable to cause the expulsion of the child, on account of a very decided anteversion of the uterus. I performed the operation of turning and extracted the child by the feet; unusual hæmorrhage supervened, which forced me to extract the placenta rapidly, and to excite the contraction of the uterus, which was in a state of collapse. This plan succeeded, the discharge being arrested in a few moments.

"In three-quarters of an hour I withdrew, leaving her to the care of the midwife; but in half an hour after my departure, the discharge reappeared in great abundance; this was stopped by syncope. The hæmorrhage again returned; and, on this occasion, left the patient in a long-continued state of very great exhaustion and syncope.

"I was again called, and returned at the moment of the first discharge. The attendants believed her dead, and indeed she was in a state of hopeless exhaustion; she had extreme pallor, with cold extremities, pulse almost, and sometimes altogether, imperceptible, obscurity of vision, and repeated syncope. I had recourse to astringent and refrigerant applications—a concentrated infusion of ergot of rye—a cordial potion, dry frictions over the skin with a brush and with flannel, at the same time applying hot cloths to the limbs. I persevered in this manner for three quarters of an hour, without obtaining the least melioration, the state of the patient, on the contrary, gradually becoming worse, and I foresaw the inevitable approach of a fatal termination. At this moment the recollection of a case by M. Nelaton decided me to attempt the transfusion of blood, although alone, and without any of the usual instruments for the performance of this delicate operation.

"I found in the house a small child's syringe, which would hold about 70 grammes of blood, having ordered in readiness hot water, vessels, and linen; a neighbor of the patient, a girl named Faget, was kind enough to consent to allow the necessary quantity of blood to be taken from her arm. Everything being arranged, I made an incision three centim. in length over the basilic vein in the right arm of the patient, in the direction of its course. I then completely isolated the vein for the extent of two centim., and passed below it a ligature to enable me to raise the vein, and to tie it upon the point of the syringe. I then divided the coats of the vein for a demicentimetre in length: two or three drops of blood only escaped. I then compressed the vessel above and below. Having bled Faget, and the blood being received in a cup, placed in a vessel full of water sufficiently hot to preserve it at its ordinary temperature, I quickly took the syringe, previously warmed, and filled it completely with the blood, forcing forward the piston so as to be quite certain that it did not contain any air, the tube of the syringe being inserted into the opening in the vein, and, having tied the ligature lightly around its point, I slowly and with care injected the blood into the vein; after having forced the piston through one-third of its course, I felt a sudden resistance, which showed me that the blood no longer penetrated the vein either from coagulation having taken place, or from some other cause; I of course, immediately ceased to press forward the piston.

"In recommencing the operation I enveloped the syringe with linen thoroughly moistened with hot water, and this time nearly all the blood which the syringe contained was injected into the vein. The whole quantity of blood introduced by the two attempts might be calculated to be about ninety grammes, without any subsequent pain or inconvenience.

"Immediately after the transfusion, respiration became more regular, the pulse stronger, the tendency to syncope suddenly ceased, and the obscurity of vision, which had been a permanent symptom, rapidly subsided.

"To keep up this improvement, after having dressed the little wound, I recommenced the use of frictions and hot cloths, besides again having recourse to rhatany and ergot of rye.

The circulation and animal heat returned by degrees, and two hours after the operation, the patient was so well that she slept for a short period, and to this sleep succeeded an unexpected melioration, which put an end to this alarming crisis.

"From this time her convalescence was rapid; the secretion of milk took place regularly. Ten days after, the patient was able to rise for an hour in the day; on the twentieth day she was completely well, and at the end of thirty days she was able to follow her usual occupations."

